# Provenance Data Management in Health Information Systems: A Systematic Literature Review

**DOI:** 10.3390/jpm13060991

**Published:** 2023-06-13

**Authors:** Márcio José Sembay, Douglas Dyllon Jeronimo de Macedo, Laércio Pioli Júnior, Regina Maria Maciel Braga, Antonio Sarasa-Cabezuelo

**Affiliations:** 1 Department of Information Science, Federal University of Santa Catarina, Florianópolis 88040-900, Brazil; douglas.macedo@ufsc.br; 2 Department of Computer Science, Federal University of Santa Catarina, Florianópolis 88040-370, Brazil; laerciopiolijr@gmail.com; 3 Department of Computer Science, Federal University of Juiz of Fora, Juiz de Fora 36036-330, Brazil; reginambvillela@gmail.com; 4 Department of Computer Science, Complutense University of Madrid (UCM), 28040 Madrid, Spain

**Keywords:** provenance data management, data provenance, provenance data, health information systems, health data, health data management

## Abstract

Aims: This article aims to perform a Systematic Literature Review (SLR) to better understand the structures of different methods, techniques, models, methodologies, and technologies related to provenance data management in health information systems (HISs). The SLR developed here seeks to answer the questions that contribute to describing the results. Method: An SLR was performed on six databases using a search string. The backward and forward snowballing technique was also used. Eligible studies were all articles in English that presented on the use of different methods, techniques, models, methodologies, and technologies related to provenance data management in HISs. The quality of the included articles was assessed to obtain a better connection to the topic studied. Results: Of the 239 studies retrieved, 14 met the inclusion criteria described in this SLR. In order to complement the retrieved studies, 3 studies were included using the backward and forward snowballing technique, totaling 17 studies dedicated to the construction of this research. Most of the selected studies were published as conference papers, which is common when involving computer science in HISs. There was a more frequent use of data provenance models from the PROV family in different HISs combined with different technologies, among which blockchain and middleware stand out. Despite the advantages found, the lack of technological structure, data interoperability problems, and the technical unpreparedness of working professionals are still challenges encountered in the management of provenance data in HISs. Conclusion: It was possible to conclude the existence of different methods, techniques, models, and combined technologies, which are presented in the proposal of a taxonomy that provides researchers with a new understanding about the management of provenance data in HISs.

## 1. Introduction

Information Systems (ISs) and Information Communication Technologies (ICTs) provide a new and innovative mechanism of communication. In cyberspace, instant messaging communication is changing lifestyles, and business happens across all industries [1]. In this sense, advances in ICT have allowed new ways of providing services in the health areas. ICTs have substantial potential to help provide high-quality health services by promoting better outcomes through access to healthcare information [1]. Thus, the importance of the various ICT technological tools aimed at human beings led to the construction of HISs, which [2] defines as “data, information and knowledge processing systems in healthcare environments”. Telemedicine is an example of a health scenario that uses HISs in an integrated manner. It has a range of medical equipment and integrated systems that generate diagnoses and treatments regardless of geographic distance, developing patient benefits, such as reduced travel or faster access to knowledge [3,4]. It is important to emphasize that telemedicine is a health scenario that uses different HIS types for the health data management process. Like any other healthcare scenario, it generates large volumes of data that need to be managed.

According to [5], the world health sectors are characterized by the increased production of data related to patient care demands. This data production increase includes hospital records, test results, and devices that are part of the Internet of Things (IoT), among other medical data. The authors also claim that we face tons of data on various aspects of our lives, especially the healthcare industry. Like any other industry, healthcare organizations are producing data at a tremendous rate, which presents many advantages and challenges at the same time. Technological advances have helped generate a large quantity of data, becoming a complex task when using current technologies, such as blockchain, cloud computing, fog computing, and artificial intelligence, among others. In this way, some research addresses the technological aspects mentioned in the perspectives of computer science and information sciences [6,7,8,9,10]. In this sense, the need to manage large volumes of data in HISs meets the use of computational strategies that address the historical processing of this data (i.e., provenance management) [11,12]. In this article, we present a Systematic Literature Review (SLR) that investigates the computational strategies adopted using methods, techniques, models, methodologies, and technologies that contribute to the provenance data management in HISs. According to [11,12], the data provenance is essential concerning the auditing, screening, and lineage of the data. It can also be considered metadata that describe the origin and the entire path taken by the cycle of the data used.

### 1.1. Motivation

In [13], the data provenance is a process that aims to provide an overview of the origin of data used by information systems. It focuses on the origin of data, especially identifying the data sources and transformations that it has undergone over time. It is related to different application scenarios. The health scenario is the focus of this work. The use of data provenance in the health context is experiencing a growing research scenario based on the most varied types of scientific experiments. The technologies applied in this area have been obtaining expressive results [14]. However, it is essential to emphasize that one of the main problems that data provenance faces is traceability. This ubiquitous problem is usually found in databases that are the result of several transformation steps. As an example, we can mention scientific databases and data warehouses [15].

Problems in data management processes can lead to data loss and privacy exposure. In the medical context, this is vital, both in terms of security and data availability, which, in many cases, are obtained in an emergency [16]. In this perspective, in the studies selected in this SLR, challenges are presented in the use of different methods, techniques, models, and methodologies related to data provenance through technological tools that can contribute to the provenance data management in HISs. It is important to emphasize the importance of managing provenance data in HISs, as they are sensitive and essential data for medical decision making, which, in fact, is one of the greatest motivations for carrying out this research. Consequently, the interoperability between HISs has also become one of the largest problems, especially in terms of health data security. In recent years, it has been shown that the secure exchange of medical information significantly benefits people’s life quality, improving their care and treatment. The interoperability of the entire healthcare ecosystem is a constant challenge, and even more with all the risks posed to the security of healthcare information [17]. In this way, problems with interoperability, tracking, and security with regard to the management of provenance data in HISs are some of the points observed in this SLR study. Thus, this article aims to answer general and specific SLR questions, and it proposes a taxonomy related to the management of provenance data in HISs that contributes to further research in this area.

### 1.2. Contributions

The contributions of this SLR are as follows: (i) An SLR that focuses on different methods, techniques, models, and methodologies related to provenance data management in HISs, which takes into account the challenges, approaches, advantages, and used technologies; (ii) A presentation of the categories that stand out with the management of provenance data from HISs, such as storage, availability, traceability, confidentiality, integrity, authenticity, and auditability; (iii) A taxonomy is proposed considering the results of the SLR. The taxonomy proposed here presents a process for the provenance data management in HISs considering four dimensions: methods, techniques, models, and methodologies, as well as different types of HISs, computational technologies used for HISs, and international standards used in HISs; (iv) A preliminary analysis of technological tools and solutions was carried out focusing on the medical systems industry that contributes to the management of provenance data in HISs.

### 1.3. General Structure of the Work

This article is organized as follows: Section 2 describes the background, and data provenance and HISs are conceptualized. In Section 3, related works and the need for this SLR are highlighted. In Section 4, we present the review methodology used to conduct the SLR and the backward and forward snowballing technique. Section 5 presents the similarities of the selected studies by the SLR, along with the backward and forward snowballing technique. In Section 6, the SLR report is presented. Section 7 presents the proposed taxonomy for managing provenance data in HISs based on the results presented. In Section 8, the data provenance in the medical systems industry is presented. Furthermore, in Section 9, threats to the validity are presented, followed by Section 10, which discusses important open-issue points of this SLR. Finally, in Section 11 and Section 12, conclusions and future works are presented, respectively.

## 2. Background

This section presents the necessary elements for the investigations of this SLR regarding the importance of managing provenance data in HISs. In the following subsections, data provenance and HISs are conceptualized.

### 2.1. Data Provenance

There are over a hundred different ways to use the term provenance [18]. Thus, it is necessary to have a clear and expansive definition of the provenance concept. The Oxford English Dictionary provides the following description for the term provenance: (i) it comes from a specific source, origin, derivation; (ii) the history or pedigree of a work of art, manuscript, rare book, etc. [19]. Provenance refers to any amount of information, comprising all the elements and their relationships, which contribute to the existence of given data. In this sense, managing provenance has gained significant attention from the research community and industry in recent decades [20].

One of the first authors to define data provenance was Buneman [21], who describes it as the origin of a data object or the process by which it reaches a database. Another definition by the same author is the documentation of the lifecycle processes of a digital object. However, the term “data provenance” refers to the origin and may also be related to data auditing, classification, and lineage [22]. Provenance information corresponds to essential metadata that describe the entities, users, and processes involved in the history and evolution of a data object [23]. Provenance can be created by software in a specific physical, digital, or application resource, or can refer to an audit process that is operated on data created purely for the provenance information [24]. It also helps determine the authenticity and consequent value of data through some questions that serve as a traceability filter. Some instances are “What?”, “When?”, “Where?”, “How?”, “Who?”, “Why?”, and “For What?”. In this sense, the principal basis of data provenance is to collect evidence about time, place, and, if applicable, the person responsible for creating, producing, discovering, or inserting the data. For example, comparative analysis techniques, expert opinions, and results of various types of scientific tests can also be used for this purpose, but what establishes the source is its documentation [11,22,25,26,27,28,29].

The data provenance also helps in reproducing several experiments, storing the data that gave rise to the research until it reached its result. Furthermore, provenance plays an essential role in tracking data stored in different repositories, showing the validity, authenticity, and integrity of the processes generated by these data. The configuration of the data provenance in the different ISs depends on computational methods. These methods must be transformed, building a qualified provenance to be retrieved, analyzed, and well founded [21,30,31,32,33].

It is also important to note that the data provenance manifests itself in three perspectives: prospective and retrospective [25] and evolutionary [34]. The prospective provenance captures the steps that must be followed to generate a specific product (e.g., a set of processes, a script). The retrospective provenance captures the steps performed by a computational task (for example, information about the environment used to derive a particular product; that is, a detailed record of the task’s execution). Other vital components of provenance are user-defined data and documentation. They are generally derived from annotation processes and are not captured automatically. This type of record becomes essential, as it contains information about the user’s decisions and observations [25]. The evolutionary provenance reflects the changes made between two executed versions of the workflow (that is, the evolution history), keeping all the changes applied throughout its lifecycle [34].

As for how data provenance works, in general, what happens is that data in one database are moved to another database when queries or programs are executed, and thus a description is made specifying the relationships between the sources involved [26]. In databases related to the health area, the strategies used to date provenance can significantly contribute to the process of managing these data to occur in the best possible way [26].

In this sense, another important point to emphasize about the data provenance are the provenance models recommended by the World Wide Web Consortium (W3C), called the PROV family [35], among other models in the literature. According to [12], the use of data provenance, regardless of the model used, provides an essential basis for assessing the authenticity of data, allowing for reliability and reproducibility. The data provenance models that stand out for the topic of this SLR article are described below.

(i). The PROV model: Defines a provenance data model to support the interoperable interchange of provenance in heterogeneous environments, such as the web. The PROV core structure relies on the definition of the entities, activities, and agents that are involved in producing a piece of data or a thing, and on how they are related by defining the following four property types: wasGeneratedBy, wasAssociatedBy, wasAttributedTo, and used [35];
(ii). PROV-O: A provenance ontology model that establishes “[…] a set of classes, properties and restrictions that can be used to represent and exchange information from sources generated in different systems and under different contexts”. PROV-O is a lightweight ontology that can be adopted in a wide range of applications [36];
(iii). OPM: An open provenance model aimed at characterizing the provenance of any “thing”, material or immaterial. It seeks to demonstrate the causal relationship between events that affect objects (digital or not), and it describes this relationship through a directed acyclic graph [37,38]. It is important to note that the OPM model was already overlaid by the PROV model;
(iv). PROV-DM: A provenance data model according to [39] that has the main function of describing the people, entities, and activities involved in the production of data. In addition, the model creates the conditions for provenance to be demonstrated and exchanged between different systems;
(v). PROV-N: A provenance notation model that is intended for human consumption. PROV-N allows serializations of PROV instances to be created compactly. PROV-N facilitates the mapping of the PROV data model to concrete syntax and is used as the basis for formal PROV semantics [40].

Therefore, it can be observed in the authors’ conceptualization regarding the contexts of data provenance and their respective models that these approaches can significantly contribute to the management of origin data in HISs.

### 2.2. Health Information Systems

HISs are increasingly used worldwide to improve hospital efficiency, quality of care, and patient satisfaction [41]. These systems, introduced in the 1960s, are used in almost all nations, depending on the technological development of the country [42]. HISs can also be conceptualized as information systems that integrate the collection, processing, communication, and use of critical information to improve the effectiveness and efficiency of health services through better management at all levels of the health sector. This system produces relevant and quality information to support the management and planning of health programs [2,43]. Health information systems (HISs) are being implemented in all aspects of health, from administration to clinical decision support systems. The generation and storage of large volumes of data in their decentralized repositories make these processes challenging when it comes to managing these data [44]. There are different types of HISs. Most of them fall into one of two main categories. The first deals with individual data records (e.g., electronic health records). The second fits into systems associated with data collection for decision making and information management, generally called health management information systems [45].

It is important to emphasize that HISs allow all information related to the patient to be computerized, providing better and more efficient health services [46]. In the context of this study, an HIS is defined as an automated means of collecting, storing, and retrieving information about people in healthcare environments. It includes patients, doctors, nurses, and other users who collect clinical and administrative data. This process is carried out independently in the same environment or in different environments at the local and national levels [47,48,49]. We highlight in the next paragraphs the main HISs in the context of the topic in question.

(i). Electronic Health Records (EHRs): EHRs describe the concept of a comprehensive, interinstitutional, and longitudinal collection of patient health and health data. This, therefore, includes data that are not only particularly relevant to the medical assessment of a subject’s treatment, but also to the subject’s health in general [50]. It is worth noticing that when describing EHRs, we always associate the use of the Health Insurance Portability and Accountability Act (HIPAA) [51], which is standard in the United States in EHRs;
(ii). Personal Health Records (PHRs): These records are often created and monitored by the patients themselves. They can be desktop-based, Internet-based, or cellular (for example, located entirely on a cell phone or handheld storage device) [52];
(iii). The Learning Health System (LHS): This system has the ability to share data and harness their potential to quickly generate knowledge and inform transformative decisions that contribute to better health. It has an infrastructure to achieve this goal at scale, combining technology, process, and policy [53];
(iv). Healthcare Monitoring Systems (HMSs): These systems monitor health in a potential field of application for wearable sensors. These wearable and environmental sensors measure health-related data in everyday user or patient environments [54];
(v). The Clinical Research Information System (CRIS): This is a type of specialized software application designed to support clinical research that can reduce the costs of research studies. The CRIS supports clinical care, collects data for research, and supports hospital operations [55];
(vi). The Hospital Information System (HIS): This is a comprehensive, integrated information system designed to manage the administrative, financial, and clinical aspects of a hospital [56]. It is further defined as an integrated electronic system that collects, stores, retrieves, and displays general patient data, such as patient information history, laboratory test results, diagnoses, billing, and other hospital procedures [56].

The information collected in these systems can also be used to predict and combat epidemics, analyze medical practice, and monitor other aspects related to healthcare and services. Some can be carried out outside hospitals to promote health efficiency, ensuring that excellent care reaches all patients in the best possible way [47]. The World Health Organization (WHO) developed a structure of the constituent components of an HIS. In this way, it interacts with other elements, producing quality information to qualify decision making and improve the population’s health status [57]. In this sense, the HIS improved the quality of patient care and reduced clinical errors, increasing the efficiency of care, significantly reducing costs and time [42,47,58]. Another important factor is improving access, management, and health information exchange with internal and external stakeholders. This contributes to making health information available and accessible, when necessary (opportunity), allowing continuity of care and support for decision-making processes [42,46,58,59]. For example, the adoption of health information technologies in conjunction with telemedicine is rapidly evolving to assist physicians, patients, nurses, and other users in HIS use [60]. However, despite the reported benefits of the HIS, implementing it does not necessarily mean increased efficiency. Some studies in the literature observed that there was variation in the adoption of these systems. This variation is associated with the low rate associated with causes by organizational and financial factors, lack of experience of health professionals, and technological knowledge [49,60]. In this context, it is important to emphasize that the adoption of HISs by health professionals is a great challenge. To be successful, the implementation of HISs needs to consider several factors. These factors include social, economic, and technical aspects, organizational failures, and even system and software problems [42,61].

While the HIS can provide many benefits, such as reducing costs and improving healthcare quality, there are also potential challenges associated with its introduction, implementation, and use [62,63]. These challenges start with the importance of training users by professionals who use HISs. Concerns are raised among healthcare providers, managers, healthcare organizations, and patients [42,47,64]. This is due to the confidentiality, security, and privacy of information, which allow measures to be established to maintain and protect the security and privacy of users in these systems [65,66,67].

Therefore, due to the large volume of data and the need to store and retrieve health data in the HIS, managing these data is an evolutionary and necessary process to contribute to security and privacy in the most diverse health scenarios. Technologies are necessary for the management of health data, contributing to the benefits of and improvements in the intervention of the various HISs.

## 3. Related Works and the Need for This SLR

In the past, very limited research was published related to the management of provenance data in HISs. However, no dedicated and detailed study on an SLR can be found covering the processes and activities involved in managing provenance data in HISs, presenting the existing methods, techniques, models, and methodologies for management provenance data, in addition to the different types of HISs, or employed computational technologies and international standards that contribute to the successful management of provenance data in HISs.

Thus, in this section, the existing research is summarized, presenting its contributions and limitations, compared with our work. As noted in the literature, most works do not follow an SLR methodology. These works are focused on a specific application domain or consider only limited aspects or those that contribute in parts to the process of managing provenance data in HISs. This in fact highlights the importance and necessity of the current study. In Table 1, it is verified whether the recent research used the protocols of an SLR, general review techniques, were in the context of HISs, or focused on the management of provenance data in HISs.

In the study [68], the authors also focus on security as one of the important factors for the communication of health devices in the Internet of Health Things (IoHT) scenario for the smooth running of health activities. As a contribution of this study, the authors emphasize the importance of safety and provenance in the IoHT, limiting themselves only to this scenario.

In the study [69], the authors report that when working with sensitive data, such as health data, security mechanisms are needed. As a contribution to the study, the authors present research that provides a broad view of the security mechanisms applied, along with Semantic Web technologies that can allow their use with health data. These studies present mechanisms that address various attributes, such as authentication, authorization, integrity, availability, confidentiality, privacy, and provenance. Although the study addresses these issues, it is limited only to health data security mechanisms.

In the study [70], the authors discuss the challenges for healthcare data management systems in terms of data transparency, traceability, immutability, auditing, data provenance, flexible access, trust, privacy, and security. As a contribution to the study, blockchain technology is discussed as promising for healthcare sectors. The study is limited to EHRs and electronic medical records (EMRs).

In the study [71], the authors focus on the positive contributions of FHIRs, including challenges, implementation, opportunities, and future applications, limiting themselves to using FHIRs only in an electronic health record (EHR) scenario.

In the study [72], the authors discuss the Internet of Things (IoT) and blockchain for the expansion of healthcare systems in relation to their scalability and consistency on a decentralized platform. As a contribution to the study, the research focused on the IoT and eHealth systems is presented that explores the application of blockchain technology in various fields of eHealthcare, and it is limited only to this purpose.

In the study [73], the authors discuss the integration and exchange of information between health organizations, presenting some tools used in this process in the contribution of this study. The proprietary way of storing electronic health records of patient history is highlighted, limiting itself to semantic interoperability only.

In [74], the study authors discuss the importance of Health Level Seven International (HL7) and Fast Healthcare Interoperability Resources (FHIRs) as the leading interoperability standards for the healthcare data exchange and clinical research process. The study contributions are focused on expanding and funding HL7–FHIR-enabled solutions for clinical research and are limited to that purpose only.

Importantly, in the content of the studies presented in Table 1, the blockchain and health devices linked to the IoHT are trends for use in health data, which, in fact, will require provenance data management processes in future health scenarios. It is also noteworthy that most of the current works presented in this section discuss the importance of the use of artificial intelligence (AI) combined with data provenance in systems linked to the health sectors. We can agree with the statement in the study [75], in which the authors argue that data provenance is important to improve AI-based systems, which is a trend for HISs to contribute to decision making. Another important point to highlight is that our SLR makes improvements in the face of the limitations presented in the studies in this section, highlighting the importance of current research.

## 4. Review Methodology

An SLR is a type of scientific research that aims to gather, evaluate, and summarize the results of multiple primary studies. This type of review also seeks to answer a set of formulated questions, using systematic and explicit methods to identify, select, and evaluate relevant research. SLRs typically collect and analyze data from included primary studies using statistical methods that summarize their results [76,77,78,79,80,81]. This study becomes necessary because those with superior methodological quality can be used in the most varied practices, among the numerous studies published on a given topic. Furthermore, as conflicting results often emerge from different studies that address the same question, individual studies rarely have sufficient statistical power to provide definitive answers [80,82]. SLRs are of great importance as a scientific research tool for decision making at much lower costs than those required for large-scale studies [79,82].

### 4.1. Review Planning

Many activities should be considered before performing an SLR. We conducted this review based on Kitchenham’s guidelines for performing SLRs in software engineering [78]. She presented a set of steps that should be considered when building an SLR. Here, we present some steps that were used in this study:

- Choice of databases and terms for search strategy;
- Strategy and criteria for selecting primary studies;
- Strategy for assessing the quality of the selection of primary studies;
- Strategy for data extraction and synthesis;
- Identification of relevant studies;
- Strategy for a summary of relevant studies.

### 4.2. Research Question Definitions

The SLR presented here can also contribute to constructing new hypotheses, evidence, and synthesizing results that aid in managing provenance data in HISs. Therefore, one of the essential processes of an SLR is the construction of research questions [76,77,78]. This study classified questions into General Research Questions (GRQs) and Specific Research Questions (SRQs). It is important to emphasize that, for the formulation of the general questions of this SLR, the mnemonic SPICE, proposed by [83], was used. The mnemonic SPICE comprises the following: (i) setting—where? (e.g., In what context are you addressing the issue?); (ii) perspective—for whom? (e.g., Who are the participants?); (iii) intervention—what? (e.g., What is being performed?); (iv) comparison—compared with what? (e.g., What are your alternatives?); and (v) evaluation—with what result? (e.g., With what (what) result? How will you measure whether the intervention was successful?).

The phases of the SPICE mnemonic adapted for this article are as follows: (i) setting: type of HIS; (ii) perspective: HIS professionals and users; (iii) intervention: storage, availability, traceability, confidentiality, integrity, authenticity, and auditability; (iv) comparison: in addition to methods, techniques, models, or methodologies for managing provenance data, other alternatives that can be compared, such as the technologies used in HISs; and (v) evaluation: results presented by using methods, techniques, models, methodologies, and technologies used to manage provenance data in HISs.

These five phases were established to ensure the quality of the returned primary studies. The general and specific question (GRQ and SRQ) posts by the SPICE mnemonic for this SLR are presented below.

General Research Questions (GRQs): GRQ1: What are the different methods, techniques, models, and methodologies used for the provenance data management in HISs?; and GRQ2: What are the challenges regarding the different methods, techniques, models, and methodologies identified in relation to the provenance data management in HISs?

Specific Research Questions (SRQs): SRQ1: Taking into account the most representative aspects regarding the provenance data management in HISs, how did these systems approach the different methods, techniques, models, and methodologies identified?; SRQ2: What are the main advantages of applying different methods, techniques, models, or methodologies for the provenance data management in HISs?; and SRQ3: What are the main technologies identified in the different methods, techniques, models, or methodologies that contributed to the provenance data management in HISs?

### 4.3. Search String Construction and Libraries

The choice of the selected databases for searching primary studies started from the assumption of greater adherence related to the theme of the study area. They were as follows: the ACM Digital Library (see http://dl.acm.org (accessed on 11 April 2023)); the IEEExplore Digital Library (see http://ieeexplore.ieee.org (accessed on 11 April 2023)); ScienceDirect (see http://www.sciencedirect.com (accessed on 11 April 2023)); SpringerLink (see http://link.springer.com (accessed on 11 April 2023)); Scopus (see http://www.scopus.com (accessed on 11 April 2023)); and Web of Science (WoS) (see http://webofscience.com (accessed on 11 April 2023)). The interval used for searches in the chosen databases was from 2010 to 2020. This time interval was stipulated considering the pre-tests carried out in the databases, which showed more results after 2010. The searched terms were limited only to the metadata (e.g., titles, abstracts, and keywords) of the articles.

The research strategy’s string concatenated the terms “Data Provenance” and “Health” to identify different methods, techniques, models, or data provenance methodologies, considering the HIS types. According to [78], variants and synonyms related to the research topic (e.g., telemedicine, eHealth, mHealth, and healthcare) were introduced for more accurate results. Finally, the Boolean logical operators (AND, OR) were used to form the following search string: “Data Provenance AND (Health OR Telemedicine OR e-Health OR m- Health OR Healthcare)”.

### 4.4. Inclusion and Exclusion Criteria

Articles in English published in journals or conferences were considered due to their relevance in computer science. Other documents, such as technical reports, dissertations, theses, and books, among others, were not selected. In this sense, the inclusion (Table 2) and exclusion (Table 3) criteria were defined for the selection of primary studies.

### 4.5. Quality Assessment Strategy

The quality of an article can be measured by its relevance and the scientific value of its content. To assess the quality of the selection of primary studies, some criteria were introduced to check whether the articles are relevant studies or not. As described by [78], these procedures are necessary to assess the quality of selected works. The evaluation of the quality of primary studies in this article consisted of the selected studies, considering the purpose of the research, contextualization, literature review, related works, methodology, results, and conclusion, according to the aims and indication of future studies. Thus, during the analysis of the primary studies and the collection of results, the criteria formulated in Table 4 were applied, allowing for a different, broader process of validation of the studies.

Quality assessment can serve as a recommendation for future research, providing information on the quality of information from each assessed study [84]. It is described in Table 5 to cover the scope of the studies, allowing us to find answers to the general and specific questions stipulated.

To assess the adequacy degree of the quality criteria, the assessment strategy proposed by [85] was adopted, allowing gradual responses from 0 (strongly disagree) to 2 (strongly agree), as shown in Table 6.

To aid in the assessment, the Likert-3 scale was adapted for each quality criterion proposed, as can be seen in Table 7.

In this sense, the quality levels of the 14 studies selected in this SLR unanimously assumed the scale “2” for the five quality criteria (QC1, QC2, QC3, QC4, QC5) based on Table 5 and Table 6.

Soon after, adapting the Likert-3 scale for each quality criteria, the quality levels were analyzed, as proposed by [86], and they are presented in Table 8.

Thus, it was possible to observe that two studies (14.3%) presented themselves as “very good,” satisfying the quality criteria in a positive way. What drew more attention was that 12 studies (85.7%) were considered “great,” which shows that the selection of studies in this SLR has a high level of studies with significant quality for the study of data provenance management in HISs. The positive percentages presented and analyzed by the Likert-3 scale proved to be favorable to qualify the studies selected, contributing to the valorization of the content presented by the studies’ authors. After these steps, data were extracted, and these data were synthesized to obtain a broader view of the theme proposed in this article.

### 4.6. Data Extraction and Synthesis Strategy

The strategy for the extraction and synthesis of the data from the retrieved studies was carried out in a structured way through the export of the documents to Mendeley (see https://www.mendeley.com/ (accessed on 11 April 2023)) to eliminate duplicate studies. For better visualization of the data, an electronic spreadsheet containing essential information was generated. To better obtain the data in the studies, they were synthesized and designed to answer the general and specific questions.

### 4.7. Primary Studies Identification

The electronic libraries already mentioned in this article to retrieve the primary studies that make up this SLR aim to cover the essential journals and conferences within computer science. Therefore, the research results still had to pass through the filters of the SLR processes and the synthesis phase of the relevant studies. The synthesis of the relevant studies to this SLR followed some steps to filter those related to methods, models, techniques, methodologies, and technologies used to manage provenance data in HISs. After the first processes described above, the first reading was carried out. This step considered only metadata (for example, title, abstract, and keywords) and inclusion and exclusion criteria. The second reading filter, which includes introductions, results, and conclusions, was performed. Thus, it was possible to select only the articles that met the previously specified selection criteria and answer the questions in this SLR.

### 4.8. Systematic Review Conduction

**Figure 1:**
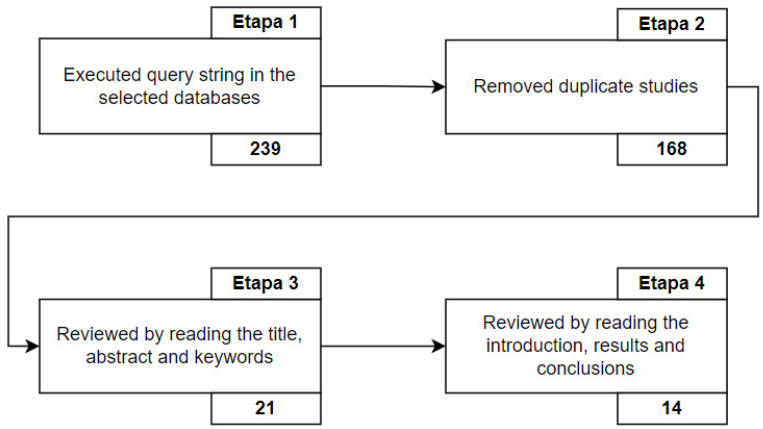
Number of studies per stage of the SLR conduction phase.

This section presents how the primary studies were identified and used to answer the questions discussed here. Figure 1 shows the selection process of primary studies at each stage of the SLR.

We observe in Figure 1 that many duplicate studies were found. This happens because digital databases often index primary studies from other databases. Different factors can justify the number of studies that are returned in each database. Some of these factors are related to the order in which the research was conducted, the total number of studies in the base, and the relevance of the base to the research question. To better understand (Figure 1), in Step 1, the query string was run on the selected databases between 25 and 27 June 2021. The search interval was 10 years (from 2010 to 2020), returning a total of 239 studies. Of these 239 retrieved studies, 11 were taken from the ACM Digital Library, 50 from IEEExplore, 3 from ScienceDirect, 66 from Scopus, 59 from SpringerLink, and 50 from Web of Science. Table 9 summarizes these data showing the number of articles retrieved per database.

In Step 2, 71 duplicate studies were found. Thus, in Step 3, the first filter was performed (reading the titles, abstracts, and keywords), in which 147 studies were discarded for not meeting the inclusion criteria of this SLR, leaving 21 studies for the execution of the second filter. It is important to emphasize that, when reading the abstracts of the studies, it was observed that they had characteristics related to the management of data from HISs. In Step 4, the second filter was performed (reading the introductions, results, and conclusions) for the remaining 21 studies. Thus, strong relationships with provenance data management in the SIS were carefully observed in 14 studies, with 7 studies showing no solid relationships and being discarded. Finally, 14 studies were selected for full reading, as they met all the selection criteria specified in the steps. For the quality assessment of the 14 primary studies selected to compose this research, we can state that all studies were evaluated following all the quality criteria already mentioned. It is important to emphasize that the exclusion process resulted in 14 studies not related to the management of provenance data in HISs. Although data provenance may have been mentioned in their abstracts as one of the use cases, it was not the focus of the authors’ research. They only mentioned data provenance in one of the subsections as a potential area of application in health, without contributing to new ideas applied in HISs.

### 4.9. Backward and Forward Snowballing

According to [78], SLRs must be executed strictly following a predefined search strategy. This search strategy must be impartial and must allow the integrity of the research to be assessed. In [78], the authors argue that initial searches for studies can be performed using several digital libraries, and they indicate that other complementary searches should be employed (e.g., manual searches in journals). An example of a manual procedure often used in addition to the SLR is snowballing. This search strategy consists of iteratively exploring the list of references (backward) and articles that have a citation of the selected article (forward) [78,87,88,89]. For this reason, we identify an initial set, defined as the starting point. This initial set is a collection of already selected studies to compose the systematic mapping, from which their references and citations will be verified [87,88]. In this set, only the studies that will be included for the final analysis are included. The next step is to start the first iteration, conducting snowballing (backward and forward). After executing the backward and forward processes, the retrieved documents are added to the total of the initial set that was evaluated at the beginning of the process [88]. The iterations are defined in [88] as follows:

- Backward snowballing: The intention is to use the study reference list to find new works to be included. When checking the list of references, exclude according to basic exclusion criteria, such as year of publication, written language, or publication type. The next step is to exclude the studies already found before, and then the others are candidates for inclusion. Then, read the other information and parts with greater relevance;
- Forward snowballing: The intention is to use the list of citations of the included works. Google Scholar can view the citations that each article has. Each citation is analyzed from an overview, and if information such as the title and abstract is sufficient, the article can be included in the list for further reading.

#### 4.9.1. Execution of Backward and Forward Snowballing

**Figure 2:**
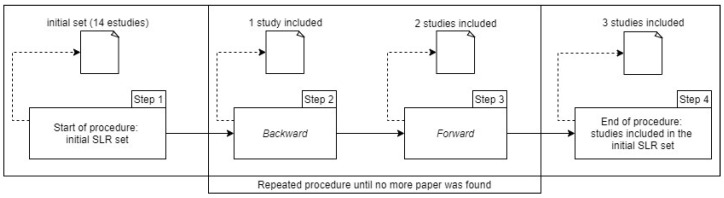
Snowballing process (backward and forward).

A second step in the SLR was performed using the snowballing technique (backward and forward) based on [88] to cover primary studies that were not previously identified. In this process, the 14 selected primary studies were used as the initial input set. It is important to emphasize that the inclusion and exclusion criteria used in the execution of the snowballing technique were the same as those used in the SLR, presented previously in Table 2 and Table 3 of this article, respectively. We considered only one caveat, in the inclusion criterion (IC1) in Table 2, in addition to studies published in articles from magazines and conferences, technical reports, and article e-books for greater breadth in the use of the execution of the technique in question here. In the execution of the snowballing technique, both backward and forward, the references were analyzed, and the primary studies that met the interests of this SLR were added to the studied scope. Figure 2 shows the steps used in the snowballing technique applied in this article.

As shown in Figure 2, the snowballing process was performed in four steps, which are described below:

- Step 1: The initial set of 14 accepted studies was evaluated to start the process of the snowballing techniques (backward and forward);
- Step 2: In the snowballing process (backward), the years of publication of the articles in the reference list were checked to see whether they met the criteria previously defined. Soon after, four verifications were carried out following the recommendations of [88]: (1) title verification in the reference list; (2) reference location verification; (3) reading of the abstract of the referenced study; and (4) verification of the complete references of the referenced study. Three iterations were performed through a manual search. In the first iteration, the proposed initial set with the references listed by the SLR was evaluated. In the second iteration, 73 studies were nominated for possible inclusion. However, in the third iteration, only 1 of the 73 studies had relevance associated with the theme of this research, and 72 were excluded. Therefore, the backward process resulted in only one new work for inclusion in the initial set, and the process was concluded;
- Step 3: In snowballing (forward), we used Google Scholar as a citation search engine. According to [88], Google Scholar avoids a bias in the search, as it indexes the main research bases among other renowned international bases. It was verified whether the year of publication of the articles also met the previously defined criteria. Soon after, four verifications were carried out following the recommendations of [88]: (1) the title of the cited study was verified; (2) reading of the study summary; (3) reading of the place of citation performed; and (4) the complete citation of the study was verified. Thus, three iterations of manual research were performed to evaluate the studies on the citation list. In the first iteration, citations from the initial set presented by the SLR were evaluated. In the second iteration, 37 articles related to the topic indicated for possible inclusion were evaluated. In the third iteration, it was observed that, in the studies found, the iterations tended to leave the initial theme increasingly dispersed, and no more relevant sources were found. Thus, the 37 studies were evaluated, and it was observed that 2 of these studies had relevance associated with the theme of this research. Therefore, 2 studies were included in the forward snowballing process, and 35 studies were excluded. No new studies were found, and the forward process was completed;
- Step 4: Finally, three studies were added to the initial set provided. In this phase, the three studies were included through the snowballing process, allowing us to delve into the topic presented in this research. It is important to emphasize that the exclusion process carried out on the backward and forward snowballing also follows the same practices described in the initial conduct of this SLR.

#### 4.9.2. Quality of the Studies Found in the Snowball Technique Process (Backward and Forward)

It is important to emphasize that the three studies found here meet the quality criteria presented in Table 4 and are considered “Great” according to the quality levels of the studies in Table 8 (both tables are already presented in this article). This shows that the quality of the studies described in this article, both in the studies resulting from SLR and in the studies resulting from the snowball technique (backward and forward), contribute to significant research on the topic in question.

## 5. Similarities of the Selected Studies

All 17 studies were found in the SLR process together with the snowball technique, which are [90,91,92,93,94,95,96,97,98,99,100,101,102,103,104,105,106] and are focused on managing provenance data in HISs. In Table 10, the similarities of the 17 studies are observed in the following characteristics that contribute to the management of provenance data in HISs: use of models from the W3C PROV family; use of different models from the W3C PROV family; use of provenance techniques with blockchain; and use of provenance techniques with middleware.

It is important to note that of the 17 studies presented in Table 10, 10 studies used models from the PROV family (that is, they focused on international standards linked to data provenance). Regarding the use of different models of the PROV family, eight studies are highlighted. This is due to the fact that they used models that are mixed depending on the technologies used. Regarding the use of blockchain technology as a way of guaranteeing the immutability of data in the provenance process, five studies stand out. Finally, in the use of middleware technology, only three studies present its use as a contributing technology for the management of provenance data in HISs.

## 6. Systematic Literature Review Report

**Figure 3:**
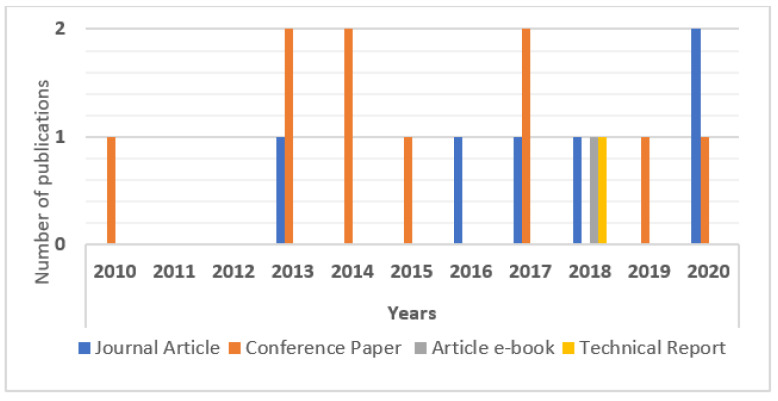
Relation of the number of studies found according to the year and type of publication.

From this section on, the 17 studies will be part of the analysis base to generate the results of this article. In Figure 3, we present the years and types of publications. The following subsections will answer the general and specific questions of this article in order to outline results that will serve for further analysis.

### 6.1. Methods, Techniques, Models, and Methodologies for Management Provenance Data in HISs

#### 6.1.1. GRQ1—What Are the Different Methods, Techniques, Models, and Methodologies Used for the Provenance Data Management in HISs?

To answer this question, Table 11 summarizes some information regarding the studies: the authors, years, type of HIS, health scenarios, and their respective method\technique\model\methodology of the data provenance in different types of HISs.

It can be seen in Table 11 that different methods, techniques, models, and methodologies for managing data from HIS provenance were presented in the selected studies. We highlight the data provenance models indicated by the W3C implemented through computational tools in different types of HISs. The models indicated by the W3C that appeared the most were as follows: PROV [91,96,98,99,103,105,106] applied in HISs (EHRs, PHRs, the LHS, the CRIS, and the HIS); PROV-O [91,95,96,98,99] applied in EHRs, PHRs, the LHS, and the CRIS; OPM [97,98,100,101] applied in HISs (PHR and LHS); PROV-DM [91,96,99,101] applied in HISs (HER, PHRs, and the CRIS); and PROV-N [98,99] applied in HISs (PHRs and the LHS). These models appear more frequently because they are application-specific models of data provenance recommended by the W3C, capable of describing the entities and processes involved in the production of a resource.

Other studies present data provenance models indicated by the W3C, combined or not, with different methods, techniques, methodologies, and technologies that enable the provenance data management in HISs. They are discussed below.

(i). Four studies presented the provenance of data using blockchain [100,102,103,104]. The use of blockchain technologies for data immutability and the use of smart contracts as a technological tool that allows for the creation of self-executing contracts that cannot be lost or tampered with are seen in these studies. In the study [104], the authors use blockchain technology to preserve the integrity of health data by permanently validating and retrieving these data in the database of a cloud PHR HIS anchored in the blockchain network. The study [100] presents a data provenance model called PROV-Chain based on blockchain technologies and the OPM model of the data provenance in an HIS of PHRs. In the study [102], the authors present the data provenance incorporated into the blockchain in an HIS of PHRs. In the study [103], the authors present the provenance data incorporated into the blockchain together with the PROV model in an HIS of EHRs. It is important to emphasize that the source data management mediated by blockchain technologies is an ally for data security in HIS scenarios. In this way, consequently, they promote the secure tracking of data and guarantee the immutability of these data in HISs;
(ii). Two other studies stand out for presenting algorithms with data provenance application techniques using middleware technology [93,94] in EHR and PHR HISs. Middleware allows the reconstruction of contextual trigger states to help the data consumer understand why the data were collected. The authors’ main idea is to share the source metadata with the data consumer (that is, any contextual information that can attest to the authenticity and accuracy of the data and assist in the interpretation of the data);
(iii). The study [90], in addition to using the algorithm with data provenance application techniques using middleware technology, combined the use of the hybrid data provenance model called TVC and ATDM. TVC uses an explicit specification of the dependency relationship between the input and output streams at each node of the processing graph on systems offering remote health monitoring services. Thus, the authors claim in their study that, by using these technologies in combination, they significantly help to improve healthcare delivery. This study was applied to a PHR HIS.
(iv). In the study [101], the authors present a data provenance model called PROV-IoT, based on the PROV-DM and OPM models in a HIS of PHRs. This model documents the history of data records considering data processing and aggregation along with security metadata to enable a foundation of trust in the source data. The model has a comprehensive structure and describes the identification of information to be included in the design of a security-aware provenance chart;
(v). The PROV-Comics model is presented in the study [99], and it is based on the PROV, PROV-DM, PROV-O, and PROV-N models in a HIS of PHRs. PROV-Comics aims to visualize data from personal health sources through comics. Each comic strip represents a specific activity, such as entering data using a smartphone app, storing or retrieving data on a cloud service, or generating a diagram from the data. Comics are automatically generated using charts from registered sources for crucial insights, such as privacy breaches;
(vi). The PTN [95] is based on the model PROV-O. The PTN is an open, global, and trusted network methodology for peer servers for the traditional web applied in a HIS of EHRs. Sites that comply with the architecture communicate information about the transactions of any sensitive data items with the PTN. These usage records are stored in a decentralized manner and can be consulted later to verify compliance with individual usage restrictions that state that no data transfers or unauthorized use have occurred;
(vii). The provenance date in the BFTRN model in the study [92] is applied together with a retroactive reasoning algorithm motivated by the theory of time automation based on this model. In this way, it lists critical alarms applied in an HMS HIS. This model efficiently calculates the value of the diffuse time function for each transition from complex and critical alarms to the health monitoring system for proper decision making. The data provenance in this model is associated with the data captured in relation to critical alarms issued by the health monitoring system.

Therefore, it is possible to observe in Table 11 that the provenance data management in HISs is not limited to the use of specific methods, techniques, models, and methodologies, but to the use of hybrid combinations of different computational technologies to achieve the expected success.

#### 6.1.2. GRQ2—What Are the Challenges Regarding the Different Methods, Techniques, Models, and Methodologies Identified in Relation to the Provenance Data Management in HISs?

After reading the primary studies, it was possible to observe that the authors recommend attention to issues related to inconsistencies, leaks, and data security that can occur in HISs, even without user intervention. These issues pointed out by the authors are considered by them as still pending challenges that need more precise computational strategies to make the provenance data safer and more reliable in the HIS. Consequently, this helps ensure the secure storage, publication, and preservation of these data as well. We also observed that there is no common structure that can guarantee total security regarding the provenance data management in HISs. One example is the creation and exchange of personal health records distributed across multiple HISs, which present technical and clinical challenges that can put patient safety at risk. Current health information sharing systems ensure the interoperability of patient records across facilities. However, they have limits for presenting physicians with the clinical context of medical record data. Therefore, we observe that there are several technical challenges for the implementation of the security of provenance data in HISs. In fact, this can promote the success or failure of the data quality, whether in providing metadata or in matters of the interoperability, privacy, and confidentiality of the data. Finally, other challenges are related to the real-time applications that occur with health devices in PHR/IoHT scenarios. Although health institutions are betting on the use of real-time applications, such as mobile health devices, which generate significant improvements in everyday life, challenges still hinder the adoption of these devices in health, such as regulatory, financial, and organizational issues, in addition to the lack of interoperability standards between HISs.

#### 6.1.3. SRQ1—Taking into Account the Most Representative Aspects Regarding the Provenance Data Management in HISs, How Did These Systems Approach the Different Methods, Techniques, Models, and Methodologies Identified?

**Figure 4:**
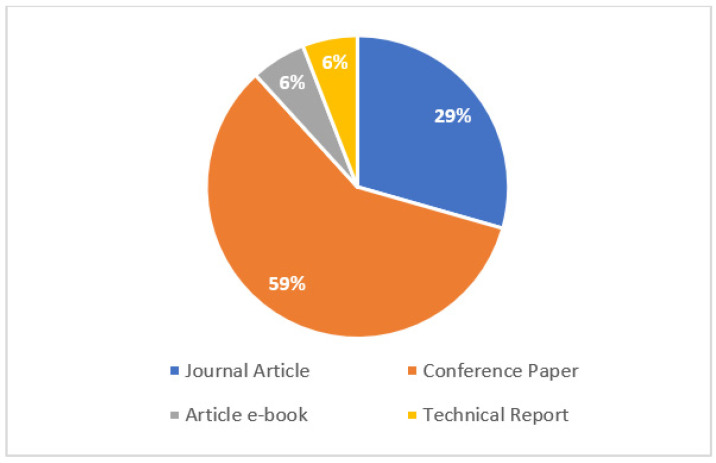
Percentages referring to types of publications.

To answer this question, it was initially observed that the number of publications each year varied among 1, 2, or 3 studies, with 2013, 2017, 2018, and 2020 being the most productive years. This may be linked to the great advance in international conferences in the field of computer science on aspects related to ICT in data provenance applications in HISs. Of the studies selected, more than half were published at conferences. In this sense, to affirm this fact, one can observe the types of publications associated with this article (journal articles, conference papers, article e-books, and technical reports), as shown in Figure 4.

**Figure 5:**
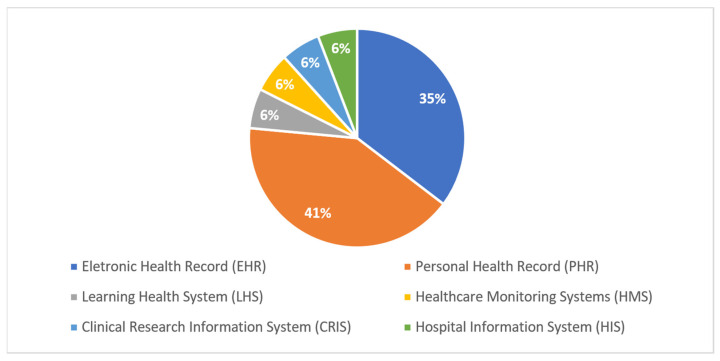
Percentages referring to the frequency of appearances of types of HISs.

Figure 4 shows that most studies (59%) were published as conference papers, while 29% of the studies were published as journal articles. Publications of article e-books (6%) and technical reports (6%) were also observed. In the health area, this happens because scientific events are a source of relevant information for knowledge sharing, allowing the dissemination of scientific evidence, good practices, and new technologies, and contributing to the improvement in and updating of research in the sector. Furthermore, the largest number of publications in the field of computing is linked to empirical studies published at international conferences, which generally have practical computational applications demonstrated in case studies. These are topics of great interest for the use of these practical applications by technology companies in the most varied health scenarios. This indicates one of the factors that have contributed to the provenance data management in relation to the representative and technical aspects that aroused interest in the use of HISs. The types of HISs that manage the provenance data through methods, techniques, models, methodologies, and computational tool technologies were also observed. The selected studies show that, as of 2010, large volumes of data began to be generated in HISs. Thus, it was necessary to implement and combine several computational strategies so that it was possible to manage the provenance data in HISs for possible decision making. Figure 5 shows, in percentages, the mentioned frequencies of the main types of HISs in the question of managing data provenance.

As shown in Figure 5, the highest frequencies of applying the provenance data management in HISs are present in the PHRs and EHRs, with 41% and 35%, respectively, followed by the HIS of the LHS (6%), HMS (6%), CRIS (6%), and HIS (6%). Considering the reading of the studies selected, these HISs presented in Figure 5 were considered the most applicable in relation to the management of origin data in the different health contexts found. It is important to emphasize that the types of HISs shown in Figure 5 have different characteristics, as detailed in this study. Moreover, based on the information in Figure 5, the authors who mentioned the types of HISs in their studies in relation to the management of provenance data in the selected studies are as follows: EHRs [93,94,95,96,103,106]; PHRs [90,97,99,100,101,102,104]; the LHS [98]; HMSs [92]; the CRIS [91]; and the HIS [105].

Another important point to emphasize is that it was possible to observe in the selected studies the exponential growth from 2010 onwards in relation to the large volumes of data generated in HISs, which is, in fact, a global trend. It was also observed that it was necessary to implement computational strategies in the use of different methods, techniques, models, and methodologies combined with computational technologies to manage the source data in HISs. It was also observed that there is a trend towards the use of computational strategies for the data provenance management in HISs considering remote health monitoring scenarios. Mobile or wearable devices coupled with the concepts of the IoT employed in healthcare represent some of these scenarios. This shows a global evolutionary trend in the provision of health services in different health settings and in different HISs for the coming years. Therefore, it can be said that the provenance data management in HISs uses and combines different computational strategies, creating a hybrid strategy to meet the needs of managing large volumes of data that contribute to decision making in these systems.

#### 6.1.4. SRQ2—What Are the Main Advantages of Applying Different Methods, Techniques, Models, or Methodologies for the Provenance Data Management in HISs?

In all the selected articles, the main advantages were observed in relation to the provenance data management in HISs: (i) maintaining the integrity of digital objects in terms of where they came from, how they were obtained, how they got to their current state, and who or what acted on them, thereby generating a greater source of trust; (ii) advantages associated with the auditability, transparency, evaluation, availability, quality, and correctness of data, generating confidence in the interoperability between HISs; (iii) increasing the visibility of the health data source and its transformations in its lifecycle; (iv) increasing health institutions’ confidence in the authenticity and confidentiality of shared data; (v) a better understanding of the sources of large volumes of health data; (vi) possibilities of using original electronic health data; (vii) retrieving new health data sources; and (viii) the importance of managing data of origin in HISs for making important decisions in these systems. It is important to remember that healthcare professionals using HISs cannot rely on or use healthcare data without knowing their original sources. This, in fact, would lead to errors that would compromise the health of their patients. Thus, based on the studies selected for analysis, we can state that the provenance data management in HISs can significantly contribute to the improvement in these systems in relation to the tracking of source data and, consequently, generate health benefits.

#### 6.1.5. SRQ3—What Are the Main Technologies Identified in the Different Methods, Techniques, Models, or Methodologies That Contributed to the Provenance Data Management in HISs?

There are a number of technologies that can be targeted towards managing provenance data in HISs. In this sense, it was possible to identify the main technologies that combine computational strategies for the provenance data management in HISs presented by the authors. The main technologies found and the respective authors that relate them are as follows: (i) The use of Extract Transform and Load (ETL), software tools whose function is to extract data from different systems, transform these data according to business rules, and finally load the data. The studies [96,98] present this technology; (ii) The studies [91,92,93,94,99,102] present mobile technologies (smartphones, tablets, notebooks, sensors for data collection) and Personal Digital Assistants (PDAs) for monitoring medical data from a distance [90]; (iii) In order to create an efficient way to represent data on the World Wide Web in order to build a global database of connected data, using the strategies of the Semantic Web Extensible Markup Language (XML) [90,94,101,105,106], Ontology Web Language (OWL) [91,96,98,105], and Resource Description Framework (RDF) [91,96,98,105], and the use of semantic web languages such as SPARQL Protocol and RDF Query Language [91,98,104]; (iv) Cloud computing structures are presented in the studies [90,94,99,100,101,102,103] as a way to guarantee the amount of resources needed for their operations to occur without errors or bottlenecks; (v) The studies [90,91,92,94,95,97,100,101,102,104] present private networks for monitoring patient data. The authors describe that these private networks contribute to the security and non-leakage of monitored patient data; (vi) To manage one or more databases, database management systems are used [91,92,93,94,95,96,97,99,100,101,102,103,104,105], and specifically MySQL [90] and Neo4j [98,99], as well as the use of a standard driven declarative query language such as the Cypher query language [98]; (vii) The studies [103,106] present the Digital Imaging and Communications in Medicine (DICOM) technology, one of the main sets of standards for the treatment, storage, and transmission of medical information in electronic format, structuring a protocol. DICOM is widely used in several HISs; (viii) The use of Character Separated Value (CSV) files is presented in the study [99], Clinical Document Architecture (CDA) is presented in [103,106], and Continuity of Care Documents (CCDs) [96] and the Portable Document Format (PDF) are used in the studies [102,103,106]; (ix) The compact, open standard independent format of simple and fast data exchange between JavaScript Object Notation (JSON) systems is used in the studies [98,103,106]; (x) Blockchain technologies are presented in the studies [100,102,103,104,106]; (xi) Middleware technologies are presented in the studies [90,93,94].

There are international standards for the representation and transfer of clinical and administrative data, and a system of standards for cataloging and sharing patient records between healthcare institutions between HISs, which are as follows: (i) the Health Insurance Portability and Accountability Act (HIPAA) [95]; (ii) Integrating the Healthcare Enterprise (IHE) [103,106]; (iii) Health Level Seven International (HL7) [103,105,106]; (iv) Fast Healthcare Interoperability Resources (FHIRs) [103,106]; and (v) Cross Enterprise Document Sharing (XDS) [103,106].

According to the authors, these technologies can be combined to manage provenance data in HISs. However, in addition to the advantages already described in SRQ2, all these technologies present implementation and interoperability challenges between different HISs. Thus, not all technologies mentioned in the studies guarantee 100% efficiency. In fact, with the advance of the large volume of data generated in health scenarios, the technological tools and strategies are being improved according to the demand and need to manage the source data in HISs. The provenance data management in HISs may depend not only on implemented technologies, but also on professionals who are well trained to use these technologies, as well as on processes and policies imposed by health institutions.

### 6.2. Main Categories Identified in Relation to the Management of Provenance Data in HISs

After reading the selected articles, it was observed that most of the authors listed, directly or indirectly, discuss seven categories that we understand as the main ones for the safety of the management of data of origin in HISs in their proposals. They are as follows: storage, availability, traceability, confidentiality, integrity, authenticity, and auditability. These categories, in fact, contribute to the provenance data management in HISs. Table 12 shows that, of the studies analyzed, the following categories were identified.

The seven categories presented in Table 12 have a context that is extremely focused on the contribution of the management processes of provenance data in HISs. These categories are explained as follows: (i) Storage: Health data stored in provenance repositories. The storage of health data in HISs is of great relevance for the traceability and availability of reusing these data; (ii) Availability: Essential to ensure that health data are available for the proper functioning of ongoing healthcare. Available health data can also contribute to medical research; (iii) Traceability: The location of health data on a data map. The traceability of health data in its various stages and processes contributes to the identification of activities within an HIS, such as participants, locations, times, and more; (iv) Confidentiality: The guarantee of the privacy of health data in HISs. The confidentiality of health data is related to access by persons expressly authorized in the HIS. In fact, it is HIS protection to prevent unauthorized people from gaining access; (v) Integrity: Ensuring the accuracy and reliability of provenance health data throughout their lifecycle. With regard to the integrity of health data, they must be retrieved in their original form (at the time they were stored). This prevents intentional or accidental unauthorized modifications to the HIS; (vi) Authenticity: The certainty that health data come from announced sources and that they have not been mutated during a process within the HIS; and (vii) Auditability: Certification of electronically stored health data repositories as to their integrity, reliability, and compliance with the laws governing the healthcare institution. Auditing health data in an HIS is a process that increases the credibility of health services, and it is also responsible for keeping legal and internal policies always up to date.

Comparing the categories presented in Table 12 with the studies from [107,108,109,110,111,112,113], we can state that these categories are part of the security requirements and use of data provenance that can be perfectly associated with applications in relation to the provenance data management in HISs. Thus, the categories listed by the authors become crucial and of great relevance to support the optimal management of the provenance data from HISs.

Therefore, it was observed that, in the large volumes of data generated by HISs, the fact of managing origin data can significantly contribute to the quality of health services offered by medical institutions. In fact, these categories positively affect decision making that requires analysis processes based on the source of data contained in the HIS databases.

### 6.3. Lessons Learned

After analyzing the articles selected in this SLR, we extracted a set of lessons learned in the process of managing provenance data in HISs: (i) It is a complex process, and it involves several computational technologies integrated into several highly heterogeneous workflows; (ii) There are a number of methods, techniques, methodologies, and data provenance models, including the models recommended by the W3C for this process to occur; (iii) International standards for the transmission of health data between HISs is essential for management process provenance data; (iv) The management of provenance data in HISs has reached unprecedented proportions. Health data from HISs are crucial to the health benefits of populations; (v) Cost reduction, the security of health data, and the centralization of data for further analysis and decision making in HISs. Driving decisions in HISs with quality data is a good approach to contribute to risk reduction and obtain satisfactory results; (vi) Managing the growing volume of provenance data in healthcare is critical for science, as this translates into knowledge generation for the healthcare sectors.

## 7. Towards a Taxonomy for Provenance Data Management in HISs

A taxonomy that involves the IS area connected to health structures can contribute to structuring the knowledge and emerging research in health information technologies. It is necessary to study the high complexity and diversity of health information technologies. Therefore, a taxonomy contributes to the identification and structural nature of constructs relevant to the development of theories in healthcare settings [114]. Although there are studies that explicitly relate some types of categorization schemes, taxonomies, and identification of a significant number of comparison dimensions for data provenance characteristics, as in the case of the studies [20,28,107,115,116,117,118], these studies do not address aspects related to the provenance of health data specifically in HISs. Although these studies make it a complex process to provide a comparison and, at the same time, identify applications and aspects related to the management of provenance data in HISs, they served as a basis for building the data provenance assumptions for the taxonomy proposed here. Therefore, it is important to emphasize that the taxonomy proposed here has an adaptive character; that is, it proposes a taxonomy related to the management of provenance data specifically in HISs, allowing it to be expanded, improved, and evaluated by other researchers in future studies in different scenarios of HISs.

It is important to point out that the provenance data management in HISs is not restricted to specific issues of provenance. Thus, from a comprehensive and systematic view, using previous and recent studies in the area, we defined a unified taxonomy to contribute to the strategies of data provenance management from different types of HISs. The proposed taxonomy is divided into four dimensions: (i) methods, techniques, models, and methodologies, for management provenance data existing in HISs; (ii) different types of HISs; (iii) computational technologies employed in HISs; and (iv) international standards between HISs. These dimensions were abstracted from the main characteristics observed in the studies already described and analyzed using this SLR. These dimensions are presented in the following subsections.

### 7.1. Methods, Techniques, Models, and Methodologies for Management Provenance Data Existing in HISs

There are several different methods, techniques, models, and methodologies in the literature that can be used to manage provenance data in HISs. In the literature of the selected studies, we can see which ones authors mention, which are as follows: (i) PROV [91,96,98,99,103,105,106]; (ii) PROV-DM [91,96,99,104]; (iii) PROV-N [98,99]; (iv) PROV-O [91,95,96,98,99]; (v) OPM [97,98,100,101,103]; (vi) PROV-IoT [101]; (vii) PROV-Comics [99]; (viii) PROV-Chain [100]; (ix) PTN [95]; (x) BFTRN [92]; (xi) TVC [90]; and (xii) ATDM [90].

### 7.2. Different Types of HISs

HISs can be observed in several countries, and their use to streamline the processes in relation to health data are observed. This dimension lists the main internationally known HISs that manage provenance data: (i) EHRs [93,94,95,96,103,106]; (ii) PHRs [90,97,99,101,102,103,105]; (iii) the LHS [98]; (iv) the CRIS [91]; (v) HMSs [92]; and (vi) HISs [105].

### 7.3. Computational Technologies Employed in HISs

This dimension presents the main computational technologies listed by the authors according to the literature of selected studies that relate the provenance data management in HISs in their proposals. The main technologies are as follows: (i) ETL [96,98]; (ii) mobile technologies (smartphones, tablets, and sensors for data collection) [91,92,93,94,99,102,103] and PDA [90]; (iii) use of the Semantic Web (XML [90,94,101,105,106]), OWL [91,96,98,105], RDF [91,96,98,105]), and semantic web languages (SPARQL [91,96,98]); (iv) cloud computing structures [90,94,99,100,101,102,103]; (v) private networks for monitoring patient data [90,91,92,94,95,97,100,101,102,103]; (vi) relational and non-relational database management systems [91,92,93,94,95,96,97,99,100,101,102,103,104,105]; use of MySQL [90] and Neo4j [98,99], and use of a standard driven declarative query language such as the Cypher query language [90]; (vii) DICOM standards set [103,106]; (viii) document type (CDA [103,106]; CCD [106]; PDF [102,103,106]; CSV [99]); (ix) JavaScript Object Notation (JSON) [98,103,106]; (x) blockchain [93,100,102,103,104,106]; and (xi) middleware [93,94].

### 7.4. International Standards between HISs

**Figure 6:**
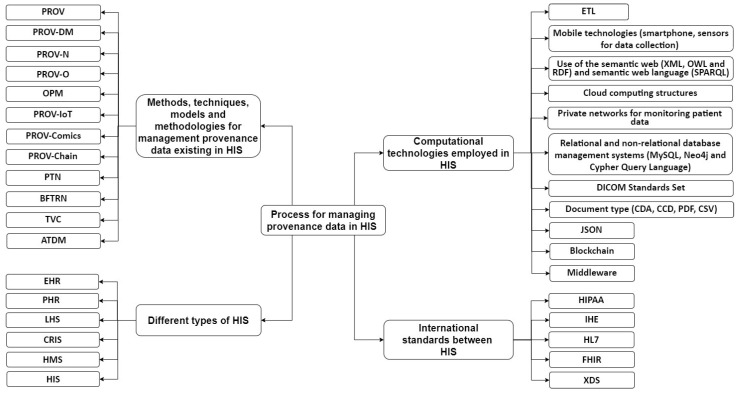
Proposed taxonomy for provenance data management in HISs.

In this dimension, the main international standards existing among HISs that contribute to the data provenance management process are listed by the authors of one of the studies: (i) the HIPAA [95]; (ii) IHE [103,106]; (iii) HL7 [103,105,106]; (iv) FHIRs [103,106]; and (v) XDS [103,106]. The proposal of our taxonomy is limited to the main classifications of a specific area (data provenance) (that is, in the context of managing data provenance in HISs). The elements of the four dimensions mentioned above were identified, considering not only those that have been widely used for the longest time, but also those that have emerged recently. The proposed taxonomy presented in Figure 6 illustrates a process for the provenance data management in HISs, covering a spectrum of alternatives along the specified dimensions.

In Figure 6, we present a proposal for a unified taxonomy for provenance data management in HISs. This taxonomy covers four dimensions observed in relation to the results of the general and specific questions based on the readings of the primary studies previously selected in the SLR. Thus, the aim of this taxonomy proposal is to guide a set of essential characteristics that contribute to the provenance data management in HISs. The main elements of each dimension existing in the proposal of our taxonomy were considered by the studies previously read in the SLR as elements of high interest for the area of data provenance in the context of HISs. Our taxonomy also considers the impact of managing the provenance data in HISs that occurs in different health scenarios more frequently. This, in fact, contributed to the observation that the use of different methods, techniques, models, methodologies, and computational technologies combined to manage provenance data is a trend to be considered in different HIS scenarios.

Therefore, given the wide variety of terms and concepts used in the literature relating to the provenance data management in HISs, we not only provide the reader with a consistent taxonomy of provenance data concepts, but also relate them to terminology used by other researchers. As a result, our taxonomy focuses on different directions regarding the flow of the provenance data management in HISs.

Finally, the four dimensions of our taxonomy aim to inform and improve the understanding of the distinction between different perspectives regarding the provenance data management in HISs. In fact, our taxonomy can contribute to the decision and selection of the most adequate solution for the needs of the healthcare scenario. In addition, potential researchers in the field, software developers, and others interested in the available approaches to managing provenance data in HISs presented here can understand the open problems seen in practice in order to improve their research and contribute to new implementations.

## 8. Data Provenance in the Medical Systems Industry

Industrial efforts in data provenance are increasingly evolving, particularly in the healthcare industry, which has aggressively invested in provenance technology [119]. In this sense, the entire healthcare ecosystem is moving towards Healthcare 4.0, through industry 4.0 methodological applications [120]. As the scope of this article is focused on the analysis of studies found in the scientific literature, we seek to follow some of the contributions of studies [121,122] to perform a preliminary analysis of the main tools or technological solutions that contribute to the management of provenance data in HISs found in the medical systems industry.

In this sense, using the five essential elements for the use of data provenance that are part of the taxonomy of [28] (e.g., data quality, audit trail, replication, attribution, and informational), five questions were elaborated to evaluate the technologies or solution technologies in the medical systems industry. These questions serve to create the characterization process based on studies [121,122]. Q01—What does the tool or technological solution offer to qualify the provenance data in HISs?; Q02—Does the tool or technological solution provide the opportunity to carry out audit tests on the provenance data to be managed in HISs?; Q03—Does the tool or technological solution make it possible to generate the replication of provenance data managed in HISs?; Q04—Does the tool or technological solution enable the attribution of provenance data managed in HISs?; Q05—Does the tool or technological solution have the informational concept in relation to provenance data managed in HISs? To answer these questions, we used Google Scholar following the criteria defined in [121]. Thus, in the eight retrieved studies, we obtained a set of 10 tools or technological solutions found in the medical systems industry that contribute to the management of provenance data in HISs. After that, to evaluate the 10 tools or technological solutions based on studies [121,122], we used the following rules: (i) Y means “Yes” and represents that this tool or technological solution fully answers this question; (ii) N means “No” and represents that this tool or technological solution does not support this question; (iii) P means “Partially” and represents that this tool or technological solution only partially supports this question. Table 13 presents the preliminary assessment of the 10 tools or technological solutions found in the eight studies referring to the medical systems industry. They are artificial intelligence (AI); big data analytics (BDA); cloud computing; fog computing; the IoT; FHIRs; findable, accessible, interoperable, and reusable (FAIR); consumer-generated health data (CGHD); HL7; and blockchain.

Regarding the technological tools or solutions evaluated in Table 13, some appear more frequently, such as blockchain, IoT, cloud computing, and fog computing. Based on the reading of the studies, evidently this frequency is related to the need to use computer networks in health and the large volume of data generated over time. In this sense, the tools or technological solutions mentioned above that appear most frequently are currently the ones that contribute most to the management of provenance data in HISs pointed out by the medical systems industry.

Another important observation is that the vast majority of studies fully answer the questions prepared based on the study by [28] on the use of data provenance impartiality. Thus, from Table 13, we can observe that most tools or technological solutions are suitable for managing provenance data in HISs. Most of the tools or technological solutions evaluated in Table 13 are contained in the studies evaluated. Finally, we consider that, with this very preliminary analysis, the current situation shows that there is an important evolution for software engineering in this aspect. This opens the way for broader future research on this topic, as the studies presented in Table 13 present relevant technological tools or solutions for the management of provenance data in HISs, which are still in constant evolution. Therefore, it is important to emphasize that the technologies/solutions presented in Table 13 go beyond the relevant studies presented in Table 11, as they present even more differentiated approaches to the problem investigated in this SLR.

However, an important point to be highlighted in the technologies/solutions presented in Table 13 is the concern with the reliability of the systems used in the management of provenance data in HISs. For [130,131], reliability plays a very important role in obtaining quality software. Analyzing the study [130,131] in the context of provenance data management in HISs, it is a necessary factor in the medical industry to guarantee the quality of stored and shared information and the reliability of health data.

## 9. Threats to Validity

In this section, we discuss the main risks that this work poses, although SLRs are generally considered reliable [132]. Based on this assumption, this SLR presents and classifies the different methods, techniques, models, and methodologies for managing data from different types of HISs, as well as the main health application scenarios with the respective computational technologies used. Although the selection of studies for this SLR was based on firmly established inclusion and exclusion criteria, its characterization is subject to interpretation bias. The chosen databases and the execution date could also be factors that lead to an interpretation bias. The proposed content of the selected studies contains different areas of research in the use of the studied topic. In fact, this sometimes makes it difficult for readers to understand and interpret the main points of connection with the theme of this article. The number of studies analyzed is also a factor that deserves attention. Even using the same inclusion and exclusion criteria defined for the SLR in this article to perform the snowballing technique (backward and forward), few studies were selected. In this sense, the review interval at first seems quite limited, as it does not cover provenance data management studies related to targeted workflows in bioinformatics, physics, and biomedical engineering, but only in HISs. This could complicate understanding and limit the scientific impact of this review to many other related domains. However, it is essential to emphasize that this does not invalidate the analyses described above. Another fact that deserves attention is that the provenance data management in HISs is always accompanied by different computational technologies in order for them to be successful. Thus, the data provenance cannot always be understood in its original concept, which opens the possibility of a characterization that does not represent the true proposal of the researcher. Another important point is about the limits and scarcity of publications on the discussed topic found in the literature due to its development in HISs. Therefore, by identifying the scientific studies for this SLR, it is expected that they will somehow contribute to the understanding and delimitation of the scope of this research regarding the management of provenance data in HISs. Finally, a preliminary analysis of studies focused on the medical systems industry was carried out to highlight some of the most current technological tools or solutions, and to complement the knowledge generated here.

## 10. Open Issues

Regardless of the methods, techniques, models, or methodologies, together with the technologies that the authors used to manage the data provenance in the different HISs presented in their studies, they faced open questions that depended on attention to adequate solutions. The main open issues presented in Table 14 were observed in the 17 studies analyzed in the SLR and in the 8 studies focused on the medical systems industry. Possible solutions to the open issues were based on the general reading of each study, absorbing the authors’ experiences. For each study mentioned in Table 14, the authors themselves highlighted the possible solution to the open issues.

## 11. Conclusions

The provenance data management in HISs is presented in different methods, techniques, models, and methodologies in different health scenarios using different computational technologies. However, this theme is still barely explored in the literature. In this SLR, we focused on studies that presented approaches in relation to the provenance data management in HISs in order to map what already exists, and to explore what is being developed in relation to this theme. Based on the results of this SLR, it was possible to answer general and specific questions. Thus, in relation to the main methods, techniques, models, and methodologies found for managing data from different HISs, it was possible to identify the models indicated by the W3C that most appeared in the studies selected for analysis, which are PROV, PROV-O, OPM, PROV-DM, and PROV-N. In addition to these models, which were observed with greater frequency of application in the management of provenance data in HISs, other models were observed based on the PROV family, such as PROV–IoT based on PROV-DM and OPM; PROV-Chain based on blockchain technologies and the OPM model; PROV-Comics based on PROV, PROV-DM, PROV-O, and PROV-N; PTN based on the models PROV-O, BFTRN, TVC, and ATDM and an algorithm with data provenance techniques for middleware. In a way, they can have different applications in different HISs, depending on the need for and use of computational strategies, which are mentioned in this SLR. Different types of HISs were found and are presented in this SLR, such as EHRs, PHRs, the LHS, HMSs, the CRIS, and the HIS, as PHRs appeared with 41% in the selected studies in this SLR. In fact, reading these studies demonstrates the appreciation of provenance data in terms of storage, availability, traceability, confidentiality, integrity, authenticity, and auditability in these systems. Special attention should be paid to EHRs, which must comply with HIPAA standards, regulated in the United States, focusing on the confidentiality, integrity, and availability of protected health information. The main benefits of the HIPAA standards in healthcare institutions are as follows: ensuring the confidentiality, integrity, and availability of all information created, received, stored, or transmitted; identifying and protecting against threats to data security or integrity; protecting data against uses or disclosures not consented to by the data subject; and ensure that employees and collaborators comply with good information security practices. Therefore, it is important that healthcare institutions, such as hospitals, seek to comply with HIPAA standards in order to be seen as institutions that meet the most rigorous international standards of health information security. This, in fact, contributes to the success of the strategies used to manage provenance data in HISs. It is noteworthy that, of the 17 studies selected for this SLR, 59% are conference papers, justifying a common situation in publications in the field of computer science. It is also important to highlight that, of the studies selected in this SLR, those dated 2020 present HISs focused on the IoT in health (IoHT) scenarios, remote health monitoring, and mobile health devices monitored in cloud applications, among other scenarios that contemplate the convenience of patients, which, in fact, present themselves as a global trend in health scenarios. It is also important to highlight that AI, blockchain, middleware, fog computing, cloud computing, BDA, and HL7 FHIRs, among other technologies, in addition to being highlighted, are trends that contribute to the management of provenance data in HISs. An important point to be highlighted is in relation to the challenges found in the studies referring to the different methods, techniques, models, and methodologies that were identified in relation to the management of provenance data in HISs, such as inconsistencies, leaks, and data security that can occur in HISs; making provenance data more secure and reliable in HISs; unusual structures with regard to security regarding the management of provenance data in HISs; limits to presenting physicians with the clinical context of medical record data; interoperability, privacy, and confidentiality issues of provenance data in HISs; and finally, challenges related to real-time applications that occur with health devices in PHR/IoHT scenarios, which may include barriers to use due to regulatory, financial, and organizational issues, in addition to the lack of interoperability standards between HISs. Another important point of observation was the identification of the main categories present in the selected studies in this SLR in relation to the management of provenance data in HISs (namely, storage, availability, traceability, confidentiality, integrity, authenticity, and auditability), which are mentioned as positive factors in the management of provenance data in HISs. In addition, by bringing together the results of the general and specific questions of this SLR, it was also possible to propose a taxonomy containing the following dimensions: methods, techniques, models, and methodologies for management provenance data existing in HISs; different types of HISs; computational technologies employed in HISs; and international standards between HISs, based on the selected studies, in order to update the understanding of the subject for researchers, software developers, and professionals working in the management of source data in HISs. Thus, we consider that the proposed taxonomy provides valuable information about the different views of the provenance data management in HISs. In addition, the taxonomy proposed here can be useful to identify similarities and differences between the technologies, methods, techniques, models, and methodologies used to manage provenance data in HISs. Another important point to highlight is related to studies focused on the medical systems industry, which present tools or technological solutions also mentioned in the studies selected in the SLR in this article. In this sense, the following stand out: blockchain, IoT, cloud computing, fog computing, and, in some studies, middleware is mentioned. In fact, this proves that the industry follows science in relation to the tools and technological solutions that contribute to the management of provenance data in HISs. In this sense, it is possible to conclude that this research presents evidence for researchers and professionals in the field to consider the necessary decision making within the HIS of their country, contemplating the benefits to healthcare.

## 12. Future Works

As for future work, we describe some perspectives: (i) We intend to improve and deepen the proposed taxonomy to make it more specific, and even implement it in a standard format, such as IMS Vocabulary Definition Exchange (IMS VDEX). In deepening the taxonomy, we also intend to insert the tools and technological solutions related to data provenance in the medical industry. By improving the taxonomy, it will be possible to organize the presentation of computational tools used in the health area in a broader way, also making it possible to present different terms in the domain of knowledge of the subject of this work, providing better discussions for knowledge generation; (ii) The PROV model recommended by the W3C stood out in most of the studies analyzed. The PROV model also stands out because it is the model that most presents characteristics based on data provenance in terms of the use of norms and standards for the interoperability of health data. In this sense, we delved into studies of this model together with fog computing and blockchain technologies, enabling a broader and more practical view for improvements in the most varied contexts related to the management of provenance data in HISs; (iii) We intend to deepen issues dealing with health data standards and laws, such as the HIPAA, including Protected Health Information (PHI); the Health Information Technology for the Economic and Clinical Health Act (HITECH); the Health Information Trust Alliance (HITRUST) Common Security Framework (CSF) that unifies security controls from federal laws (such as the HIPAA and HITECH); Health Level Seven (HL7); the Canada Health Infoway; HEASNET in Japan; and ISO/TC 215 and CEN/TC in Europe [133], among other standards and laws that fall within the scope of health information security, listing the compliance needs and related resources for HISs, and to relate, in addition to the cited standards, other standards with the PROV model and its derivations, as well as to seek which data provenance models best fit the use of these standards in HISs; (iv) We intend to deepen the scope of the analysis of the SLR of this article, so that it is also possible to consider technological solutions or tools from the medical systems industry; describe a more comprehensive view of data provenance in the medical systems industry sector by presenting a more detailed analysis based on the study [134]; present the main tools or technological solutions currently used and recommended by the medical systems industry, making a counterpoint to the technologies that contribute to the data provenance in HISs, and performing new comparisons and approaching characteristics based on the study presented in [121,122]; (v) We intend to perform the identification of relationships between data provenance and blockchain in HISs, using the study of the authors of [135] as a basis, going beyond the relationships found by the authors of this study; (vi) It is intended to further deepen this SLR in the context of the use of artificial intelligence used in data provenance systems that contribute to HISs, promoting the visualization of provenance data linked to the direction of data science.

## Figures and Tables

**Table 1:** Comparison of related works on provenance data management in HISs.

References	Year	Systematic Literature Review	General Techniques Review	HIS Context	Focused on Provenance Data Management in HISs
[68]	2021	✓	✗	✗	✗
[69]	2021	✓	✗	✓	✗
[70]	2021	✗	✓	✓	✓
[71]	2021	✓	✗	✓	✗
[72]	2022	✗	✓	✓	✗
[73]	2022	✓	✗	✗	✗
[74]	2022	✗	✓	✗	✗
Our	2022	✓	✓	✓	✓

**Table 2:** Inclusion Criteria.

Criteria ID	Definition of the Inclusion Criteria
IC1	Studies published in journals and conference articles.
IC2	Studies that indicate primary or secondary experimental or theoretical approaches, where examples of applications or descriptions of experiments of real cases are presented using different methods, techniques, models, methodologies, and technologies for managing provenance data, considering the type of HIS.

**Table 3:** Exclusion Criteria.

Criteria ID	Definition of the Exclusion Criteria
EC1	Duplicate studies in the searched bases.
EC2	Studies that are not in the English language.
EC3	Studies with less than six pages.
EC4	Studies that do not refer to any type of method, technique, model, methodology, or technology for managing provenance data in HISs.
EC5	Studies that do not meet the research questions.

**Table 4:** Quality Criteria.

Criteria ID	Quality Criteria
QC1	The aims of the study must be aligned with the provenance data management in HISs.
QC2	The study should present theories or applications for managing provenance data in HIS contexts.
QC3	The study must present the proposal or experiment regarding the methods, techniques, models, methodologies, and technologies for managing provenance data in HISs.
QC4	The study must present results relevant to the use of provenance data management theory or applications in HISs.
QC5	The study must present the conclusion linked to the research aims.

**Table 5:** Related study sections.

Chapter	Description	Research Questions
Title	Title of specific study	GRQ1, GRQ2
Summary	Study summary	GRQ1, GRQ2
Keyword	Text content words	All research questions
Introduction	Problem to be solved	All research questions
Development	Concepts related to the proposal	All research questions
Methods	Scientific methodology	All research questions
Results	Results of the evaluations	All research questions
Discussion	Quantified data compared to the literature	SRQ1, SRQ2, SRQ3
Conclusion	Findings related to aims and hypotheses	SRQ1, SRQ2, SRQ3

**Table 6:** Scale of Likert-3.

Evaluation Strategy	Evaluation Criteria Using the Likert-3 Scale
Strongly agree (2)	It should be granted in the event that the paper presents in the text the criteria that fully address the issue.
Neutral (1)	It must be granted in the event that the work does not make it clear whether or not it addresses the issue;
Strongly disagree (0)	It must be granted in the event that there is nothing in the job that meets the criteria of the question.

**Table 7:** Scale for each quality criterion.

Criterion	Scale
QC1	2—Defines the aims of the study with the research context.
	1—Defines the aims but does not make the research context clear.
	0—It does not define the aims.
QC2	2—Theories and applications are presented in the study.
	1—Only one of the characteristics is reported.
	0—The study does not relate theory to practice.
QC3	2—The study presents the proposal and experiment.
	1—The study presents only one of the characteristics.
	0—The study does not present any of the characteristics.
QC4	2—The results are fully adherent to the aim of the study.
	1—The results are partially adherent to the aim of the study.
	0—No result is achieved.
QC5	2—The conclusions are clearly presented.
	1—The conclusions are not clear.
	0—No conclusions are presented.

**Table 8:** Quality levels of studies.

Low <26%	Average 26–45%	Good 46–65%	Very Good 66–85%	Great >86%	Total
0	0	0	2	12	14
~0%	~0%	~0%	~14.3%	~85.7%	100%

**Table 9:** Studies found in the chosen international electronic databases.

Databases	Returned Articles
ACM Digital Library	11
IEEExplore	50
ScienceDirect	3
Scopus	66
SpringerLink	59
Web of Science	50

**Table 10:** Similarities found in selected studies.

References	Year	Use of Models from the W3C PROV Family	Use of Different Models from the W3C PROV Family	Use of Provenance Techniques with Blockchain	Use of Provenance Techniques with Middleware
[103]	2020	✓	✗	✓	✗
[102]	2020	✗	✗	✓	✗
[101]	2020	✓	✓	✗	✗
[100]	2019	✓	✓	✓	✗
[105]	2018	✓	✗	✗	✗
[106]	2018	✓	✗	✓	✗
[99]	2018	✓	✓	✗	✗
[104]	2017	✗	✗	✓	✗
[98]	2017	✓	✓	✗	✗
[97]	2017	✗	✓	✗	✗
[96]	2015	✓	✗	✗	✗
[95]	2014	✓	✓	✗	✗
[94]	2014	✗	✗	✗	✓
[93]	2013	✗	✗	✗	✓
[92]	2013	✗	✓	✗	✗
[91]	2013	✓	✗	✗	✗
[90]	2010	✗	✓	✗	✓

**Table 11:** Final list of 17 selected studies.

References	Authors	Years	Types of HISs\Health Scenarios	Methods, Techniques, Models, and Methodologies
[103]	Margheri et al.	2020	EHRs\Healthcare	PROV and data provenance incorporated into the blockchain.
[102]	Rahman et al.	2020	PHRs\IoHT	Data provenance incorporated into the blockchain.
[101]	Jaigirdar, Rudolph, and Bain	2020	PHRs\IoHT	PROV–IoT based on PROV-DM and OPM.
[100]	Gong, Lin, and Li	2019	PHRs\IoT	PROV-chain based on the OPM and data provenance incorporated into the blockchain.
[105]	Kock-Schoppenhauer et al.	2018	HISs\Healthcare	PROV
[106]	Massi et al.	2018	EHRs\Healthcare	PROV and data provenance incorporated into the blockchain.
[99]	Schreiber and Struminksi	2018	PHRs\Health Monitoring	PROV-Comics based on the models PROV, PROV-DM, PROV-O, PROV-N
[104]	Liang et al.	2017	PHRs\mHealth	Data provenance incorporated into the blockchain.
[98]	Curcin et al.	2017	LHS\Healthcare	PROV, PROV-O, PROV-N, OPM
[97]	Sun, Lu, and Gu	2017	PHRs\Healthcare	OPM
[96]	Ramesh et al.	2015	EHRs\Healthcare	PROV, PROV-O, PROV-DM
[95]	Seneviratne and Kagal	2014	EHRs\Healthcare	PTN based on the model PROV-O
[94]	Lomotey and Deters	2014	EHRs\mHealth	Algorithm with data provenance techniques for middleware
[93]	Prasad et al.	2013	EHRs\mHealth	Algorithm with data provenance techniques for middleware
[92]	Wang and Hu.	2013	HMS\Health Monitoring	BFTRN
[91]	Kovalchuk et al.	2013	CRIS\Healthcare	PROV, PROV-O, PROV-DM
[90]	Chowdhury, Falchuk, and Misra	2010	PHRs\Health Monitoring	TVC, ATDM, and algorithm with data provenance techniques for middleware

**Table 12:** Main categories that contribute to the provenance data management in HISs.

References	Storage	Availability	Traceability	Confidentiality	Integrity	Authenticity	Auditability
[103]	✓	✓	✓	✗	✓	✗	✓
[102]	✓	✓	✗	✗	✓	✗	✓
[101]	✓	✓	✗	✓	✓	✓	✗
[100]	✓	✗	✓	✓	✓	✗	✗
[106]	✓	✓	✗	✗	✗	✗	✗
[106]	✓	✓	✗	✓	✓	✓	✗
[99]	✓	✓	✓	✗	✓	✗	✗
[104]	✓	✗	✗	✗	✓	✗	✗
[98]	✓	✓	✓	✗	✗	✓	✓
[97]	✗	✗	✗	✗	✓	✗	✗
[96]	✗	✓	✗	✗	✗	✗	✗
[95]	✓	✓	✗	✓	✓	✓	✓
[94]	✓	✓	✓	✓	✓	✓	✓
[93]	✓	✓	✗	✓	✗	✓	✓
[92]	✗	✓	✓	✗	✓	✓	✓
[91]	✗	✓	✓	✗	✗	✗	✓
[90]	✓	✓	✗	✓	✗	✗	✗

**Table 13:** Assessment of the characterization of tools or technological solutions.

References	Types of HIS\Health Scenarios	Tools\Technological Solutions	Q01	Q02	Q03	Q04	Q05	Totals		
[120]	EHRs\Healthcare	AI	Y	Y	Y	P	Y	Y: 4	N: 0	P: 1
[123]	EHRs\Healthcare	HL7	P	Y	P	P	P	Y: 1	N: 0	P: 4
[124]	PHRs\IoTH	CGHD	P	Y	P	Y	Y	Y: 3	N: 0	P: 2
[125]	EHRs\Healthcare	FHIRs	P	Y	P	Y	Y	Y: 3	N: 0	P: 2
[126,127]	EHRs, PHRs\Healthcare	Fog computing	P	Y	Y	Y	Y	Y: 4	N: 0	P: 1
[120,127]	EHRs, PHRs\Healthcare	BDA	P	Y	Y	Y	Y	Y: 4	N: 0	P: 1
[128]	EHRs\Healthcare	FAIR\FAIR4Health	P	Y	P	Y	Y	Y: 3	N: 0	P: 2
[120,129]	EHRs, PHRs\Telehealth, Telemedicine and Healthcare	Blockchain	Y	Y	Y	Y	Y	Y: 5	N: 0	P: 0
[120,126,127]	EHRs, PHRs\Healthcare	IoT	P	Y	Y	Y	Y	Y: 4	N: 0	P: 1
		Cloud computing	P	Y	Y	Y	Y	Y: 4	N: 0	P: 1

**Table 14:** Possible solutions to the open issues of the studies analyzed in the SLR and in the medical systems industry.

Open Issues	Reference	Possible Solutions
Confidentiality, availability, portability, integrity, security, privacy, storage, and health data leakage	[94]	Three-tier architecture using cryptography and packaging of medical data in mobile health devices that power the EHRs. Middleware-oriented cloud source data storage.
	[95]	Development of PETS that makes transparency a key component in system architectures. PETS is based on open web standards and features PTN. An open, global, and trusted network of peer servers. The use of United States HIPAA standards when focusing on confidentiality, integrity, and availability of health information, valuing the three main pillars of information security.
	[99]	Use of data visualization technique through information in comic book format, representing a specific activity, such as data entry using a smartphone application that stores and retrieves data in the cloud service, generating a diagram of the data.
	[120,126,127]	Cloud computing and fog computing in IoT scenarios were solutions that offered agility and security.
	[100,104,120,129]	Use of immutable properties of blockchain technology in the use of smart contracts within PHRs and EHRs. Use of decentralized blockchain technologies.
	[120]	Use of artificial intelligence.
	[101]	Use of security metadata for provenance across all layers of the IoHT environment.
	[102]	Decentralized consensus mechanisms based on blockchain technologies in decentralized repositories.
	[103]	No recording of any personal data directly on the blockchain, ensuring their availability. Only individuals who already have a health document (and are therefore authorized by the EHR) can query and retrieve the provenance of the corresponding blockchain.
	[124]	The use of CGHD by mobile health devices in IoT and IoT health.
Interoperability between HIS	[103,123,125,128]	Use of the efforts of international consortia, such as IHE, HL7, FHIRs, and FAIR, that have led to ubiquitous and affordable services in different facilities. In addition, several legacy integrity protocols are used, such as secure email exchange via the gateway.
	[106]	Use of blockchain technology and smart contracts along with international standards, such as IHE, HL7, and FHIRs, allowing data source queries for all types of clinical information from anywhere.
Data Reliability	[96]	Provenance structure for mHealth devices with middleware techniques. This consists of collecting and sharing provenance metadata to help the consumer verify that specific provenance properties are satisfied with the data they receive.
Data integration and analysis	[96,120,127]	Use of semantic web technologies to support large-scale secondary analysis of health data. Combination of semantic provenance with ontology-based reasoning, big data analytics.
Data inference	[90]	Collection of personal health data through algorithms with techniques of data origination in physiological sensors to be transported back to middleware through a cell phone.
Quality of data capture	[91]	The pervasive computing–powered computerized data collection approach with data provenance techniques can significantly improve the quality and eliminate data entry errors in the collection of clinical data.
	[97]	Use of only data provenance techniques to assess the quality of electronic health data.
	[98]	Use of semantic web techniques.
	[105]	Capture of interoperable provenance data needed by data administrators to assess health data that are reused in a research context.
Uncertain processes and fuzzy transformation time	[92]	Use of the BFTRN model combining semantic provenance and time-spread data from the Petri net.

## Data Availability

Data available on request due to restrictions eg privacy or ethical.
